# An Unfortunate Case of Tuberculous Meningitis in a 25-Year-Old Primigravida in the Postpartum Period

**DOI:** 10.7759/cureus.87220

**Published:** 2025-07-03

**Authors:** Deepika Kashyap

**Affiliations:** 1 Obstetrics and Gynaecology, All India Institute of Medical Sciences (AIIMS) New Delhi, New Delhi, IND

**Keywords:** extra-pulmonary tuberculosis (eptb), paradoxical response, post-partum fever, premature rupture of membranes (prom), tuberculous meningitis (tbm)

## Abstract

Tuberculosis remains a significant global health concern, with substantial diagnostic challenges in extrapulmonary tuberculosis, particularly in vulnerable states like pregnancy and the postpartum period. The postpartum period is associated with significant immunomodulation that can predispose women to reactivation or progression of tuberculosis. A healthy 25-year-old woman who presented to the obstetrics and gynaecology department with premature rupture of membranes, delivered vaginally while being on conservative management. In the postpartum period, she developed high-grade fever with chills and headache, which progressed to altered sensorium and diplopia. The patient underwent imaging and lumbar puncture after informed consent and was diagnosed with tubercular meningitis. TB can present with non-specific symptoms. Recognition of risk factors for TB is crucial for prompt diagnosis and treatment of this deadly disease.

## Introduction

Tuberculosis (TB) remains one of the world’s leading causes of morbidity and mortality in low- and middle-income countries, accounting for an estimated 1.25 million deaths in 2023 [1]. Each year, approximately 10.6 million new TB cases occur globally, of which an estimated 33 % are among adult women [2]. Of all the TB patients, 15% have extrapulmonary TB, and among them, 5-10% affect the central nervous system (CNS) [3]

Pregnancy is a period of relative immune-compromised state because of immunomodulation, nutritional stress, and hormonal changes, along with stress from sleep deprivation in the postpartum period. Commonly, TB flare-ups are seen in pregnancy because of systemic immune modulation with a complex balance of immunosuppression to prevent fetal rejection and maintenance of the ability to fight off infections, with a switch from cell-mediated immunity to humoral immunity [4]. This shift in immunity and rapid reversal of these changes in the postpartum period may result in a flare-up of TB [5].

Here, we present a case of tuberculous meningitis (TBM) that presented antenatally with non-specific symptoms of occasional headache and vomiting, and finally, in the postpartum period, developed into high-grade fever, which finally progressed to altered sensorium, hemiparesis, and ptosis. Finally, with the help of imaging and lumbar puncture, TBM was diagnosed.

## Case presentation

A 25-year-old primigravida, who conceived with in-vitro fertilisation for tubal factor infertility in her sixth attempt, presented to the labour suite at 22 weeks of gestation with pre-term pre-mature rupture of membranes. She was a known case of hypothyroidism. At presentation, there were no complaints of abdominal pain, fever, or bleeding per vaginum. Her antenatal period was fairly unremarkable except for a history of threatened abortion in the first trimester. On examination, her general condition was good. Her vitals were stable. Systemic examination was normal. On per-abdomen examination, the uterus corresponded to a 20-week size, relaxed, non-tense, and non-tender. On per speculum examination, rupture of the membrane was confirmed. Pros and cons regarding the continuation of a previable pregnancy were discussed with the patient and relatives. Neonatal prognostication was done. The patient insisted on conservative management. Hence, intravenous antibiotics were given for 48 hours, followed by oral erythromycin as per standard hospital protocol.

The patient was observed for chorioamnionitis based on clinical and laboratory parameters, including vital observation, abdominal and local examination. She was given a steroid course at 24 weeks of gestation for fetal lung maturity. However, there were complaints of on-and-off headaches and vomiting during her antenatal period, for which physician consultation was sought, and migraine was suspected and managed conservatively. She had a history of diagnostic hystero-laparoscopy with bilateral (B/L) tubal clipping in 2020. Laparoscopy revealed that the omentum adhesions, hydrosalpinx, and flimsy adhesions were present all around the uterus. She went into spontaneous labour at 25+4 weeks period of gestation and delivered a female baby vaginally, which was followed by manual removal of placenta under general anaesthesia.

**Figure 1:**
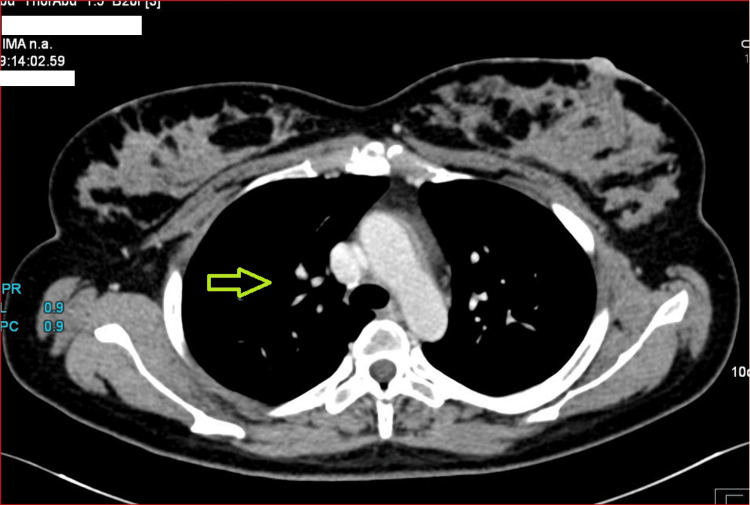
Patchy consolidation in apicoposterior segment of left upper lobe

On postpartum day 3, the patient developed high-grade unremitting fever with chills with no localising signs of infection. Blood culture, urine culture, high vaginal swab and placental membrane culture were sterile. COVID-19 reverse transcription polymerase chain reaction (RT-PCR) and tests for influenza, typhoid, malaria, and dengue were negative. Ultrasound pelvis showed an empty uterine cavity with no features of endometritis. As routine investigations were negative, contrast computed tomography (CT) was performed. High-resolution CT chest (Figure 1) showed multiple centrilobular nodules in the lung, predominantly in the upper lobe, suggestive of tuberculosis

**Figure 2:**
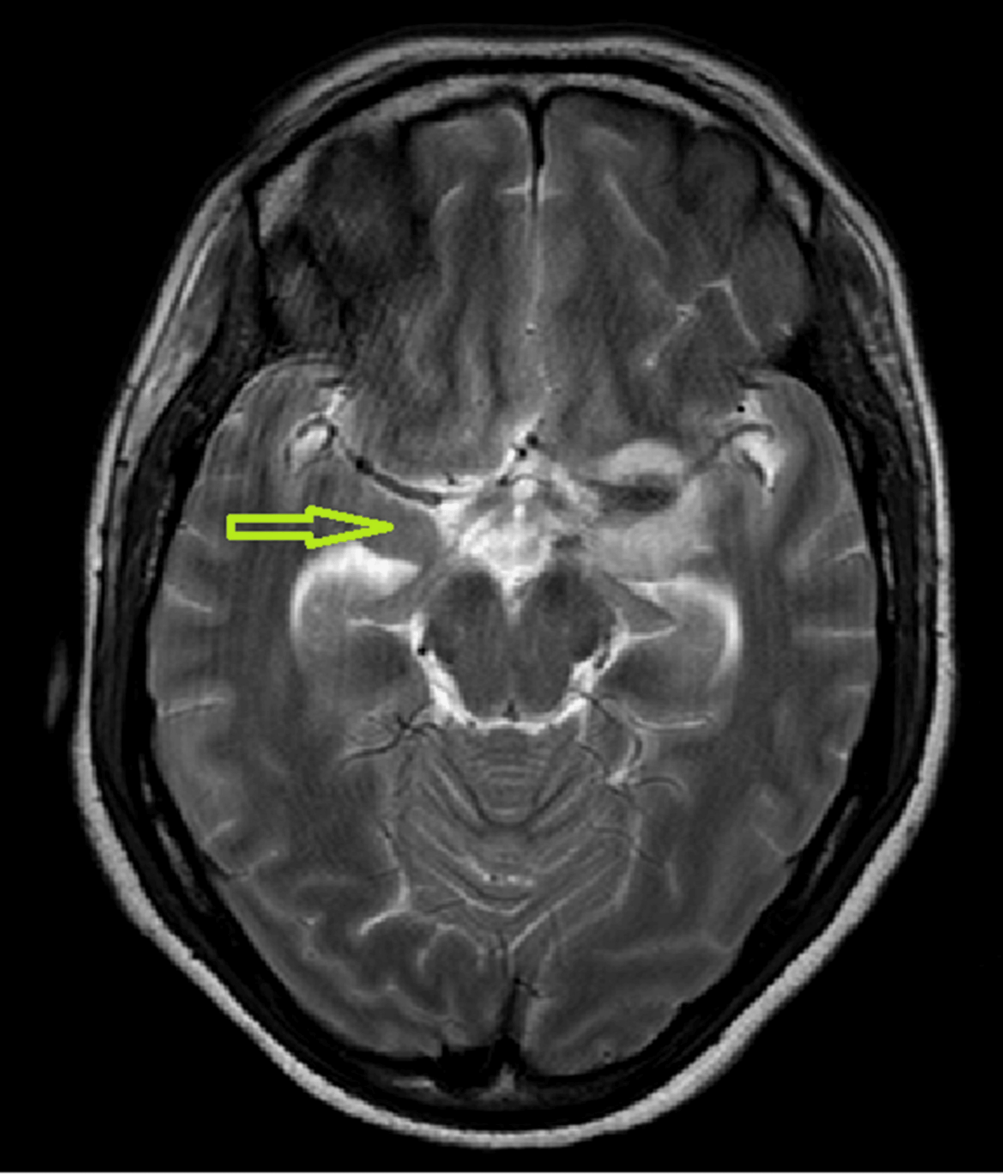
Axial MRI T2 image showing basal exudates and leptomeningeal enhancement

Pulmonary consultation was sought based on CT findings and a clinically deteriorating condition. The patient was shifted to the pulmonary intensive care unit because of atypical pneumonia. On postnatal day 8, she developed altered sensorium, right-sided weakness, and left eye ptosis. As advised by the neurologist, contrast-enhanced magnetic resonance imaging (MRI) of the brain and lumbar puncture were performed after informed consent. Axial T2-weighted MRI of the brain showed an area in the basal cisterns that was hyperintense on T2 (yellow arrow, Figure 2), suggestive of exudates or inflammation, which was highly suggestive of basal meningitis.

**Figure 3:**
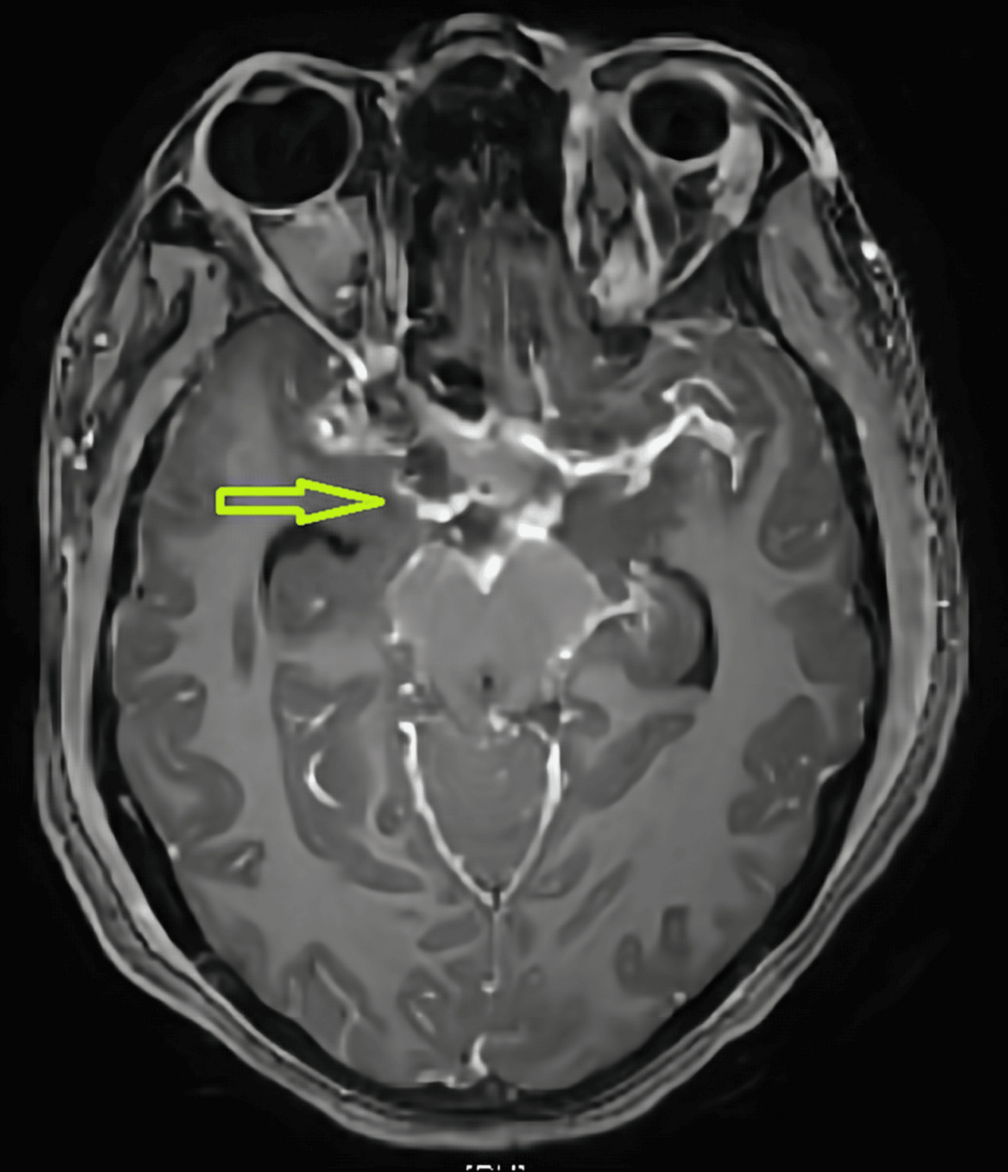
Axial T1-weighted post-contrast (gadolinium-enhanced) MRI image showing lepto-meningeal enhancemnt

The patient was shifted to neurology for further management. Neurological examination showed a Glasgow Coma Scale (GCS) score of E4V5M6, accompanied by drowsiness. The left eye had ptosis with complete ophthalmoplegia involving cranial nerve III, IV, and VI. The left eye pupil was sluggishly reacting, and right upper motor neuron (UMN) seventh cranial nerve palsy was observed. Fundus examination revealed bilateral grade I papilledema. The patient had neck rigidity and Kernig's sign among the meningeal signs. During motor system examination, hypotonia was noted in the right upper and lower limbs with power of 0/5. Deep tendon reflexes were exaggerated with the right plantar extensor reflex. MRI brain revealed leptomeningeal enhancement (Figure 3), and lumbar puncture was positive in the Xpert MTB/RIF (Cepheid, Sunnyvale, California, United States) with elevated adenosine deaminase (ADA) and lactate dehydrogenase (LDH).

Based on the results, TBM was diagnosed, and the patient was started on weight-based anti-tubercular drugs comprising isoniazid, rifampicin, pyrazinamide, and levofloxacin, which was subsequently changed to streptomycin, given a doubtful episode of seizure observed by attendants, along with pyridoxine. The patient was also started on weight-based injectable steroid in the form of pulse methyl prednisolone, initially followed by injectable dexamethasone for two weeks. It was gradually tapered to oral steroids along with Ecosprin. In the recovery period, the patient was given Ryle’s tube feed due to altered sensorium, bladder and bowel care, along with physiotherapy.

During her recovery in the hospital, she had improvement in sensorium, overall improvement in well-being, became afebrile, and was discharged with modified Rankin Score (mRS) 4 status. At the two-month follow-up after ATT, non-contrast CT and MRI were repeated to observe the evolution of the previous changes. Susceptibility-weighted imaging (SWI) MRI was suggestive of blooming in the peri-sylvian area.

Since the patient had a very preterm delivery, the baby had respiratory distress syndrome and received surfactant within the first week of life. During follow-up, the baby developed a grade 2 intraventricular haemorrhage with two episodes of clinical sepsis requiring antibiotics. In the neonatal intensive care unit, the baby was intubated for 10 days, followed by non-invasive ventilation for a month, followed which the baby was again intubated with high ventilator settings attributed to severe bronchopulmonary dysplasia. The baby tested negative for TB. However, unfortunately, the baby died on the 55th day of life with neonatal sepsis.

## Discussion

TB during pregnancy and the postpartum period is a growing concern, with an estimated 200,000 women affected globally each year, including approximately 151,000 during pregnancy and 49,000 in the postpartum period [6]. The risk of TB is increased by 1.3-1.4 times during pregnancy and nearly doubles (IRR ~1.9-2.0) in the postpartum period due to immunological changes [1].​​​​ The incidence of tuberculosis is higher in pregnant females as compared to non-pregnant females because of the immunomodulation seen in pregnancy and the post-partum period [7]. In this report, we discussed a young female who presented with preterm premature rupture of membranes with non-specific symptoms of headache and with post-partum flare of neurological signs and final diagnosis of TBM. Pregnancy likely increases the risk of reactivation of latent TB, as seen in similar cases [8-10]. CNS involvement, primarily in the form of TBM, occurs in approximately 5-10% of extrapulmonary TB cases, with recent large-scale studies reporting rates around 7% [11]. However, because of its devastating consequences and nonspecific symptoms, prompt recognition and timely diagnosis are needed to improve the prognosis and survival of the patient.

The Lancet scoring system can be used to diagnose TBM in patients based on clinical features, CSF analysis, and imaging [12]. In our patient, the score was >6, which is suggestive of TBM. Once TBM is suspected, nucleic acid amplification test (NAAT) can be used to aid in rapid diagnosis. Common NAAT tests that can be used are Xpert and Xpert-ultra, which have almost 100% specificity for TBM but have only 50-60% sensitivity [13]. If TBM is suspected, empirical treatment can be started with anti-tubercular therapy (ATT) to improve patient prognosis, as late treatment can increase mortality for the patient. In our patient, CSF Gene Xpert was positive, and hence, ATT was started with weight-based four-drug therapy. During the treatment with ATT, patients can develop a paradoxical response due to a disproportionate inflammatory response after initiation of ATT. While the exact mechanism is unclear, high antigenic load and immune reconstitution after mycobacterial death have been proposed to drive the inflammatory response in paradoxical response [14]. Corticosteroids can be used to decrease this inflammatory response and, hence, improve the survival rate in TBM patients [15]. We started our patient on weight-based injectable steroid (pulse MPS followed by injectable dexamethasone for two weeks) to cater for this paradoxical response.

TB can have an effect on pregnant women as well as the fetus. The known effects on pregnancy are risk of abortions, anaemia, fetal growth restriction, increased risk of preterm birth, pre-eclampsia, six-fold increase in perinatal death, vertical transmission, postnatal transmission, and increased maternal mortality [16]. In a study by Yadav et al., obstetrical and perinatal outcomes were seen in women with extra-pulmonary TB [17]. Retrospective data in pregnant women with extra-pulmonary TB were collected by them over 10 years, and it was observed that out of a total of 30 women, 22 were already on ATT, and eight were detected during pregnancy; 15 had genital TB, seven had bone TB, three had CNS TB, three had lymph node TB, one had intestinal TB, and one had disseminated TB. There was a significant increase incidence of oligohydramnios, preterm rupture of membrane, and preterm labour, with one maternal mortality and one stillbirth [17].

The WHO recommends that pregnant women living in areas of high TB burden (a prevalence of 100 cases per 100,000 population or greater) should be screened for TB at every contact with a health worker, using the four-symptom screening method consisting of cough, night sweats, fever, and weight loss [18]. Screening for weight loss should not just consider absolute weight loss, but should also check for failure to adequately gain weight during pregnancy. Thus, it is crucial to have an in-depth assessment during antenatal visits.

## Conclusions

Pregnancy and the postpartum period are associated with flare-ups and delayed diagnosis of TB. Hence high index of suspicion is required as the risk of untreated active TB disease to the pregnant woman and the fetus is greater than the risk of treatment. Timely diagnosis and management improve the prognosis significantly.
